# Preliminary in vitro effects of trehalose, pentoxifylline and MitoTEMPO on post-thaw quality of ram semen

**DOI:** 10.1186/s13028-026-00882-5

**Published:** 2026-09-04

**Authors:** Caner Öztürk, Mustafa Bodu, Ali Erdem Öztürk, Mustafa Numan Bucak, Neşe Hayat Aksoy, Ramazan Arslan, Mehmet Bozkurt Ataman

**Affiliations:** 1 Department of Reproduction and Artificial Insemination, Faculty of Veterinary Medicine, Aksaray University, Aksaray, 68100 Türkiye; 2 Department of Reproduction and Artificial Insemination, Faculty of Veterinary Medicine, Selçuk University, Konya, 42003 Türkiye; 3 Department of Reproduction and Artificial Insemination, Faculty of Veterinary Medicine, Erciyes University, Kayseri, 38039 Türkiye; 4 Department of Biochemistry, Faculty of Veterinary Medicine, Aksaray University, Aksaray, 68100 Türkiye

**Keywords:** Acrosome integrity, Antioxidant capacity, Freezing-thawing, Oxidative stress, Semen extender

## Abstract

**Background:**

Ram semen cryopreservation is associated with reduced post-thaw sperm quality, partly due to membrane destabilization and oxidative stress. This preliminary in vitro study compared trehalose, low-dose pentoxifylline, and MitoTEMPO, used individually or in combination, as additives for ram semen cryopreservation. Semen collected from six healthy Merino rams during the breeding season was pooled on each collection day and divided into eight treatment groups: control, trehalose (50 mM), pentoxifylline (0.25 or 0.5 µM), MitoTEMPO (0.025 or 0.05 mM), and two MitoTEMPO plus pentoxifylline combinations. This procedure produced eight pooled ejaculates per treatment group. After freezing and thawing, sperm motility, plasma membrane integrity, acrosome integrity, capacitation status, total antioxidant capacity, and total oxidant status were evaluated.

**Results:**

Trehalose and pentoxifylline at 0.5 µM significantly improved several post-thaw sperm quality traits. Compared with the control group, trehalose significantly increased post-thaw motility from 31.25 ± 4.43% to 42.50 ± 5.35%, plasma membrane integrity from 53.78 ± 2.47% to 65.29 ± 4.14%, and acrosome integrity from 54.66 ± 4.41% to 69.69 ± 1.48% (*P* < 0.05). Pentoxifylline at 0.5 µM also resulted in higher motility (40.63 ± 4.96%), plasma membrane integrity (64.24 ± 2.09%), and acrosome integrity (64.56 ± 2.69%) than the control group (*P* < 0.05). The overall treatment effect was significant for total oxidant status (*P* = 0.037) and total antioxidant capacity (*P* < 0.001). However, Sidak-adjusted pairwise comparisons did not identify significant differences between individual treatment groups for total oxidant status. MitoTEMPO alone did not significantly improve the main post-thaw sperm quality traits compared with the control group, and the MitoTEMPO plus pentoxifylline combinations did not provide a significant additional benefit. Capacitation status did not differ significantly among groups (*P* = 0.178).

**Conclusions:**

Trehalose and pentoxifylline at 0.5 µM improved several in vitro post-thaw sperm quality traits and total antioxidant capacity of cryopreserved ram semen. Because the study used pooled ejaculates and did not include fertility trials, these findings should be interpreted as preliminary and require confirmation using individual ejaculates, objective sperm motion analysis, and fertility outcomes.

## Background

Cryopreservation of ram semen is a valuable tool for genetic preservation and artificial insemination. However, cryopreservation decreases post-thaw sperm quality and may compromise fertility potential. The freeze-thaw process induces stress in sperm cells, including ice crystal formation, osmotic shock, and oxidative damage [1]. This results in the excessive generation of reactive oxygen species (ROS) during cryopreservation, inducing lipid peroxidation in the sperm plasma membrane and adversely affecting membrane integrity [2].

Recent studies have focused on supplementing semen extenders with various cryoprotective additives to mitigate cryodamage. Trehalose, a non-permeating disaccharide sugar, is an effective extracellular cryoprotectant for spermatozoa. Previous studies have reported that trehalose stabilizes cell membranes during dehydration and cooling, mitigating osmotic stress and protecting cells against ice crystal damage [3, 4]. The supplementation of freezing media with trehalose has been reported to improve post-thaw sperm motility, membrane integrity, and viability by exerting protective effects on the sperm membrane and mitigating oxidative damage [4, 5]. Trehalose may also prevent premature capacitation or acrosome reactions during the freeze thaw process. In ram spermatozoa, trehalose suppresses lysophosphatidylcholine-induced acrosome loss, thereby enhancing sperm cryosurvival [6].

Frozen semen quality can also be improved using metabolic stimulants and antioxidants, such as pentoxifylline (PTX). PTX, a methylxanthine derivative and phosphodiesterase inhibitor, upregulates intracellular cyclic adenosine monophosphate (cAMP) levels in sperm, enhancing ATP availability and sperm motility [7]. Recent studies have reported that PTX enhances post-thaw sperm function in several species. However, the degree of improvement varies depending on the dose and application stage. PTX promotes the recovery of sperm motility and exerts beneficial effects on sperm cell membrane and mitochondrial stability. For example, the supplementation of the freezing extender with PTX improved post-thaw motility of buck semen [8], as well as frozen thawed bovine and canine semen [9, 10]. In equine semen, the application of PTX after thawing stimulates sperm motility, suggesting its ability to support short-term functional activation [11]. Recent studies on human sperm cryopreservation have reported that PTX improves the viability of limited sperm samples before freezing [12].

Animal studies have indicated that MitoTEMPO supplementation can improve sperm quality. MitoTEMPO is a mitochondria-targeted antioxidant designed to reduce mitochondria-derived oxidative stress. It consists of a triphenylphosphonium moiety, which facilitates mitochondrial accumulation, linked to a TEMPO nitroxide group with superoxide scavenging activity [13]. In ram semen, MitoTEMPO enhanced the quality of chilled semen throughout the storage period. Notably, the 5 µM concentration improved motility and viability and was accompanied by a reduction in lipid peroxidation. Collectively, these results suggest that MitoTEMPO may serve as a useful additive for preserving semen quality during chilling [14]. Furthermore, the combination of mitochondria-targeted antioxidants and conventional cryoprotectants can potentially exert synergistic beneficial effects on post-thaw sperm quality. The co supplementation of ram or goat semen extenders with mitochondria-targeted antioxidants, such as MitoQ or MitoTEMPO, and trehalose yielded superior post-thaw sperm quality and fertility outcomes when compared with the supplementation of single additives alone [15, 16].

Although trehalose, PTX, and MitoTEMPO have each shown potential as cryoprotective supplements, they have mostly been studied separately. As a result, their relative effects under identical ram semen cryopreservation conditions remain unclear. We hypothesized that trehalose, low-dose PTX, and MitoTEMPO would improve in vitro post-thaw sperm quality and oxidative balance parameters, and that combination treatments might provide additional effects compared with single-additive supplementation. The present preliminary in vitro study therefore aimed to compare these additives, used individually or in combination, with particular emphasis on post-thaw structural integrity and oxidative balance.

## Methods

### Animals, semen collection and study design

This study was conducted using six clinically healthy adult Merino rams aged 2–3 years that were maintained at the university research and application farm under standard husbandry and nutritional conditions. The rams were clinically healthy, sexually mature, and had previous reproductive activity in the flock; however, detailed individual breeding history and fertility records were not available. Semen was collected twice weekly over a 4 week period during the breeding season using a sterilized artificial vagina pre-warmed to 44–45 °C. Immediately after collection, ejaculate volume, mass motility, sperm concentration, motility, and morphology were evaluated. Only ejaculates meeting the following criteria were included in the study: mass motility ≥ 3 on a 0–5 scale, sperm concentration ≥ 2.5 × 10⁹/mL, motility ≥ 70%, and abnormal morphology ≤ 15%. Eligible ejaculates were pooled to minimize individual variation among rams and to provide sufficient semen volume for simultaneous allocation to all treatment groups under identical experimental conditions.

A Tris-based extender was prepared with minor modifications to previously established formulations and contained Tris (297 mM), citric acid (96 mM), fructose (82.66 mM), penicillin G (100 IU/mL), and streptomycin (1 mg/mL). Fresh egg yolk (15% v/v) was added to the stock solution, which was then centrifuged at 3400 g for 15 min. Glycerol was subsequently added to a final concentration of 6% (v/v), and this base extender was used for all treatment groups. The study was designed to compare the effects of trehalose, pentoxifylline (PTX), and MitoTEMPO, used alone or in combination, on the post-thaw quality of ram semen. The trehalose concentration (50 mM) was selected based on previous studies in ram semen cryopreservation reporting beneficial post-thaw effects at this concentration or within a comparable range. The PTX concentrations (0.25 and 0.5 µM) were selected to evaluate whether low-dose supplementation could improve post-thaw function while minimizing the risk of excessive stimulation during cryopreservation. The MitoTEMPO concentrations (0.025 and 0.05 mM; 25 and 50 µM) were selected according to previous studies in ram chilled semen and cryopreserved sperm from other species in which micromolar-range supplementation was associated with antioxidant or sperm-protective effects [2, 14, 29]. On each collection day, the pooled semen sample was divided equally into eight treatment groups: control, trehalose (50 mM), PTX (0.25 µM), PTX (0.5 µM), MitoTEMPO (0.025 mM), MitoTEMPO (0.05 mM), MitoTEMPO (0.025 mM) + PTX (0.5 µM), and MitoTEMPO (0.05 mM) + PTX (0.5 µM). All additives were incorporated into the extender before cryopreservation. This procedure was repeated on eight separate occasions, resulting in eight independent pooled ejaculates for each treatment group (*n* = 8). Each pooled ejaculate was considered one biological replicate. After freezing and thawing, sperm motility, plasma membrane integrity, acrosome integrity, capacitation status, total antioxidant capacity (TAC), and total oxidant status (TOS) were assessed under identical experimental conditions. All animal procedures were approved by the Experimental Animals Production and Research Center Ethics Committee of the Faculty of Veterinary Medicine, Selçuk University (SÜVDEMEK) (Decision No. 2022/150, 27 December 2022) and were performed in accordance with European Directive 2010/63/EU and the ARRIVE guidelines.

### Cryopreservation procedure

After dilution, semen was loaded into 0.25 mL French straws, which were sealed using polyvinyl alcohol powder. The straws were then equilibrated at 5 °C for 2 h. After equilibration, they were positioned in liquid nitrogen vapour (-110 °C to -120 °C) approximately 4 cm above the nitrogen surface for 15 min, and subsequently immersed in liquid nitrogen for long term storage.Please check and confirm if the section headings are set correctly.

### Thawing and sperm evaluations

The straws were thawed in a 37 °C water bath for 30 s. For each replicate, three straws per group were thawed and evaluated. For each pooled ejaculate × treatment combination, the values obtained from these three straws were averaged to generate a single replicate-level value for statistical analysis. Post-thaw sperm motility was evaluated using phase-contrast microscopy under standardized conditions. An aliquot of the thawed semen (5 µL) was placed on a prewarmed slide maintained at 37 °C using a heated stage. Motility was assessed at 400× magnification to ensure accurate visualization of both general movement patterns and progressive forward motility. To minimize observer related variation, all motility evaluations were performed by the same experienced operator who was blinded to treatment groups throughout the analysis. For each sample, at least five randomly selected microscopic fields were examined. Additionally, at least 200 spermatozoa were counted per evaluation. Motility (%) was recorded based on the proportion of motile spermatozoa exhibiting any type of forward or non-forward motion.

### Plasma membrane integrity

Sperm viability was assessed using a dual fluorescent SYBR 14/propidium iodide (PI) staining protocol. Briefly, the diluted semen (30 µL) was incubated with 6 µL SYBR 14 and 2.5 µL PI in a microtube at 37 °C for 15 min in the dark. The reaction was terminated using Hancock’s solution. An aliquot of the stained suspension (3 µL) was placed on a glass slide, covered with a coverslip, and examined under a fluorescence microscope (Axioscope 5 microscope (Carl Zeiss, Jena, Germany) equipped with an Axiocam 208 colour camera) at 200× magnification. For each sample, at least 200 spermatozoa were evaluated in randomly selected microscopic fields. Viable spermatozoa with intact plasma membranes exhibited a bright green fluorescence signal in the head region, whereas non-viable spermatozoa with damaged membranes exhibited a red fluorescence signal [17].

### Acrosome integrity

The acrosome integrity was assessed using fluorescein isothiocyanate-conjugated peanut agglutinin (FITC-PNA) and PI fluorescence staining. The sperm suspension (60 µL) was incubated with 10 µL FITC conjugated lectin and 2.5 µL PI in a 37 °C water bath for 15 min in the dark. The reaction was terminated using Hancock’s solution. At least 200 sperm cells were examined under a fluorescence microscope (Axioscope 5 microscope (Carl Zeiss, Jena, Germany) equipped with an Axiocam 208 colour camera) at 200× magnification to evaluate acrosomal status. Spermatozoa exhibiting a distinct green fluorescence signal over the acrosomal region were classified as acrosome reacted or damaged, whereas those lacking FITC-PNA staining were considered to have intact acrosomes [18].

### Capacitation status

Capacitation status was assessed using the chlortetracycline (CTC) fluorescence assay, based on the method originally described by Ward and Storey and later adapted for ram spermatozoa, with minor modifications. The CTC working solution (750 µM) was prepared in a buffer containing 20 mM Tris HCl, 130 mM NaCl, and 5 mM L cysteine (pH 7.8) and kept protected from light until use. Semen samples were gently mixed with the CTC solution and processed in the dark at room temperature. The stained cells were then fixed with 4% paraformaldehyde, mounted on glass slides, covered with coverslips, and sealed to prevent drying. Spermatozoa were examined under a fluorescence microscope (Axioscope 5, Carl Zeiss, Jena, Germany) equipped with an Axiocam 208 colour camera. Based on the characteristic distribution of CTC fluorescence over the sperm head, cells were classified as non-capacitated, capacitated, or acrosome reacted. For each sample, at least 200 spermatozoa were evaluated in randomly selected microscopic fields, and the proportion of sperm displaying a capacitated CTC pattern was recorded [19, 20].

### Total antioxidant capacity (TAC)

Spermatozoa were washed twice with phosphate buffered saline (PBS). The samples were then homogenized by sonication on ice to achieve complete cellular disruption while minimizing heat related artefacts. Total antioxidant capacity (TAC) was determined using a commercial colorimetric assay kit (Baran Medical, Turkey) according to the manufacturer’s instructions. This assay is based on the automated spectrophotometric method described by Erel, in which antioxidant molecules reduce the ABTS radical cation, and the change in absorbance is measured at 660 nm. Absorbance was recorded using a Humalyzer 3000 semi-automatic photometer (Diagnostics, Wiesbaden, Germany). TAC values were expressed as µmol Trolox equivalent/L [21].

### Total oxidant status (TOS)

For total oxidant status (TOS) analysis, sperm suspensions prepared under the same conditions were evaluated using a commercial colorimetric assay kit (Baran Medical, Turkey) in accordance with the manufacturer’s protocol. The assay is based on the automated colorimetric method described by Erel, in which oxidants present in the sample oxidize ferrous ion to ferric ion under acidic conditions, and the resulting colour change is measured spectrophotometrically at 530 nm. Absorbance was measured using the same Humalyzer 3000 semi-automatic photometer (Diagnostics, Wiesbaden, Germany). TOS values were expressed as µmol H₂O₂ equivalent/L [22].

### Statistical analysis

The normality of model residuals was assessed using the Shapiro–Wilk test. All descriptive values were expressed as mean ± standard deviation. Each pooled ejaculate obtained on a separate collection occasion was considered one biological replicate. For each pooled ejaculate × treatment combination, the values obtained from three thawed straws were averaged before statistical analysis to generate a single replicate-level value. Because each pooled ejaculate was divided into all treatment groups, data were analysed using a linear mixed-effects model, with treatment included as a fixed effect and pooled ejaculate/replicate included as a random effect. This model was used to account for the shared biological origin of treatment groups within each replicate. When the overall treatment effect was significant, pairwise comparisons among treatment groups were performed using Sidak-adjusted post hoc comparisons. The P values reported in the tables represent the overall fixed effect of treatment for each parameter. A value of *P* < 0.05 was considered statistically significant. Statistical analyses were performed using IBM SPSS Statistics (version 27.0; IBM Corp., Armonk, NY, USA).

## Results

### Effect of additives on post-thaw motility

Post-thaw sperm motility differed significantly among the groups (*P* < 0.001; Table 1). Trehalose (50 mM) and pentoxifylline (0.5 µM) showed significantly higher motility than the control group (*P* < 0.05). In contrast, MitoTEMPO alone did not produce a significant change in motility compared with the control (*P* > 0.05). The MitoTEMPO + PTX combinations did not provide a significant additional motility benefit compared with trehalose or PTX (0.5 µM) alone (Table 1).

**Table 1:** Effects of additives on post-thaw ram sperm quality traits

Groups	Motility (%)	Plasma membrane integrity (%)	Acrosome integrity (%)	CTC index (%)
Control	31.25 ± 4.43ᶜ	53.78 ± 2.47ᶜ	54.66 ± 4.41ᶜᵈ	52.30 ± 2.45ᵃ
MitoTEMPO 0.05 mM	31.86 ± 4.58ᶜ	54.18 ± 2.91ᶜ	59.33 ± 3.95ᵇᶜ	52.89 ± 1.72ᵃ
MitoTEMPO 0.025 mM	32.50 ± 4.63ᶜ	56.73 ± 4.17ᶜ	59.76 ± 2.84ᵇᶜ	53.85 ± 2.22ᵃ
Pentoxifylline 0.25 µM	35.00 ± 2.67ᵇᶜ	62.36 ± 2.35ᵃᵇ	62.35 ± 2.39ᵇ	55.04 ± 2.13ᵃ
Pentoxifylline 0.5 µM	40.63 ± 4.96ᵃᵇ	64.24 ± 2.09ᵃ	64.56 ± 2.69ᵃᵇ	54.31 ± 2.12ᵃ
Trehalose 50 mM	42.50 ± 5.35ᵃ	65.29 ± 4.14ᵃ	69.69 ± 1.48ᵃ	54.83 ± 2.62ᵃ
MitoTEMPO 0.05 mM + Pentoxifylline 0.5 µM	35.62 ± 3.20ᵇᶜ	57.84 ± 2.93ᵇᶜ	52.06 ± 3.71ᵈ	53.14 ± 2.06ᵃ
MitoTEMPO 0.025 mM + Pentoxifylline 0.5 µM	33.13 ± 2.59ᶜ	57.96 ± 2.50ᵇᶜ	55.48 ± 6.21ᶜᵈ	53.45 ± 1.69ᵃ
*P* value	< 0.001	< 0.001	< 0.001	0.178

Values are presented as mean ± standard deviation from eight independent pooled ejaculates per treatment group (*n* = 8). The P value represents the overall fixed effect of treatment from a linear mixed-effects model, with treatment included as a fixed effect and pooled ejaculate/replicate included as a random effect. Different superscript letters within the same column indicate significant differences among treatment groups based on Sidak-adjusted pairwise comparisons (*P* < 0.05). Groups indicate the additive or additive combination incorporated into the semen extender before cryopreservation; the control group received the base extender without additional additive. CTC, chlortetracycline; MitoTEMPO, mitochondria-targeted TEMPO antioxidant

### Effect of additives on plasma membrane integrity

Plasma membrane integrity differed significantly among the groups (*P* < 0.001; Table 1). Trehalose (50 mM), PTX at 0.5 µM, and PTX at 0.25 µM showed significantly higher plasma membrane integrity than the control group. In contrast, MitoTEMPO alone and the MitoTEMPO + PTX combinations did not differ significantly from the control group (Table 1).

### Effect of additives on acrosome integrity

Acrosome integrity differed significantly among the groups (*P* < 0.001; Table 1). Trehalose (50 mM), PTX at 0.5 µM, and PTX at 0.25 µM showed significantly higher acrosome integrity than the control group. MitoTEMPO alone did not differ significantly from the control group. The MitoTEMPO + PTX combinations did not differ significantly from the control group (Table 1).

### Effect of additives on capacitation status

Capacitation status, assessed by the CTC index, did not differ significantly among the groups (*P* = 0.178; Table 1). Therefore, no treatment-related difference in capacitation status was interpreted under post-thaw conditions.

### Effect of additives on total oxidant status and total antioxidant capacity

Total oxidant status (TOS) showed a significant overall treatment effect (*P* = 0.037; Table 2). However, Sidak-adjusted pairwise comparisons did not identify significant differences between individual treatment groups. Therefore, no group-specific differences in TOS were interpreted.

**Table 2:** Effects of additives on total oxidant status and total antioxidant capacity in cryopreserved ram semen

Groups	TOS (µmol/L)	TAC (µmol/L)
Control	5.08 ± 3.06	344.28 ± 83.04ᶜ
MitoTEMPO 0.05 mM	3.12 ± 1.31	608.77 ± 270.96ᵃᵇᶜ
MitoTEMPO 0.025 mM	3.88 ± 2.65	564.07 ± 249.78ᵃᵇᶜ
Pentoxifylline 0.25 µM	3.63 ± 2.39	586.86 ± 198.17ᵃᵇᶜ
Pentoxifylline 0.5 µM	2.09 ± 0.71	714.74 ± 247.23ᵃᵇ
Trehalose 50 mM	2.01 ± 0.89	844.59 ± 233.54ᵃ
MitoTEMPO 0.05 mM + Pentoxifylline 0.5 µM	3.52 ± 1.49	432.90 ± 141.03ᵇᶜ
MitoTEMPO 0.025 mM + Pentoxifylline 0.5 µM	4.25 ± 1.98	595.89 ± 164.04ᵃᵇᶜ
*P* value	0.037	< 0.001

Values are presented as mean ± standard deviation from eight independent pooled ejaculates per treatment group (*n* = 8). The P value represents the overall fixed effect of treatment from a linear mixed-effects model, with treatment included as a fixed effect and pooled ejaculate/replicate included as a random effect. For TAC, different superscript letters within the same column indicate significant differences among treatment groups based on Sidak-adjusted pairwise comparisons (*P* < 0.05). For TOS, no superscript letters are shown because Sidak-adjusted pairwise comparisons did not identify significant differences between individual treatment groups despite the significant overall treatment effect. Groups indicate the additive or additive combination incorporated into the semen extender before cryopreservation; the control group received the base extender without additional additive. TOS, total oxidant status; TAC, total antioxidant capacity; MitoTEMPO, mitochondria-targeted TEMPO antioxidant

Total antioxidant capacity (TAC) differed significantly among the groups (*P* < 0.001; Table 2). Trehalose (50 mM) and PTX at 0.5 µM showed significantly higher TAC values than the control group. This was supported by Sidak-adjusted pairwise comparisons. The other supplemented groups did not differ significantly from the control group.

## Discussion

In this study, trehalose, PTX, and MitoTEMPO influenced post-thaw ram sperm quality under the present experimental conditions. Trehalose and PTX at 0.5 µM significantly improved several post-thaw sperm quality traits compared with the control group. Trehalose and PTX at 0.5 µM significantly improved motility, plasma membrane integrity, acrosome integrity, and TAC, while none of the treatments produced a significant effect on capacitation status.

Trehalose is a non-permeating disaccharide that is widely used as an extracellular cryoprotectant because of its ability to stabilize membranes during cooling and dehydration. The present findings support this protective role, as trehalose was associated with significantly higher plasma membrane integrity, acrosome integrity, and TAC compared with the control group. These results are in line with previous studies showing that trehalose can reduce oxidative damage and improve post-thaw sperm quality in different species. In rabbit sperm, for example, trehalose supplementation reduced ROS and MDA levels and improved membrane and acrosome integrity after thawing [23]. In boar semen, trehalose containing extenders improved post-thaw motility, membrane integrity, acrosome integrity, and fertilizing capacity [24]. Taken together, these findings suggest that the beneficial effect of trehalose is not limited to sperm movement alone, but also involves broader structural and oxidative protection during the freeze-thaw process.

The significant improvement in acrosome integrity observed in the trehalose group deserves particular attention. The acrosomal region is highly sensitive to cryoinjury, and damage to this structure may compromise fertilizing ability even when motility is preserved. Previous ram studies have shown that trehalose can help maintain sperm ultrastructure during cryopreservation, including acrosomal preservation [25]. In addition, trehalose has been reported to suppress lysophosphatidylcholine- induced acrosomal damage in ram sperm, which may contribute to improved cryosurvival [6]. In the present study, the absence of significant differences in the CTC index also suggests that trehalose did not promote detectable premature capacitation like changes after thawing. This point is important because a cryoprotective additive should ideally preserve sperm structure without triggering early functional destabilization.

PTX is a methylxanthine derivative and phosphodiesterase inhibitor that may enhance sperm function by increasing intracellular cAMP levels and supporting ATP dependent motility. In the present study, PTX at 0.5 µM significantly improved post-thaw motility, plasma membrane integrity, acrosome integrity, and TAC. This finding is generally consistent with previous studies showing that PTX can improve post-thaw sperm performance, although its effect appears to depend on dose, species, and the stage of application. In equine semen, a short-term improvement in motility in cooled stallion sperm has been reported, although prolonged exposure was less favourable for some sperm characteristics [26]. In goats, PTX improved several post-thaw sperm parameters, either alone or in combination with trehalose [8]. PTX has also been reported not to alter tyrosine phosphorylation patterns or membrane integrity in frozen thawed stallion epididymal sperm, suggesting that its use does not necessarily induce premature capacitation like changes [27].

MitoTEMPO is a mitochondria-targeted antioxidant designed to reduce mitochondrial ROS production. Previous studies have reported beneficial effects of MitoTEMPO on post-thaw sperm quality in different species. In human sperm, supplementation with MitoTEMPO improved post-thaw motility, viability, membrane integrity, mitochondrial membrane potential, and antioxidant enzyme activity, while reducing MDA levels [28]. In rabbits, MitoTEMPO improved motility, viability, mitochondrial activity, and acrosome integrity, and reduced ROS and apoptosis after cryopreservation [29]. In rams, MitoTEMPO improved the quality and reproductive performance of chilled semen, particularly at 5 and 50 µM [14]. More recently, MitoTEMPO, alone or in combination with MitoQ, improved several sperm quality traits and fertility related outcomes during ram semen preservation [16].

In contrast, MitoTEMPO alone did not significantly improve the main post-thaw sperm quality traits compared with the control group. Sidak-adjusted pairwise comparisons did not support a significant TAC difference between the MitoTEMPO-only groups and the control group. This may indicate species or protocol specific differences in response, or suggest that the selected doses were not optimal under the present experimental conditions. Recent studies also support this interpretation, showing that the effect of MitoTEMPO on sperm cryopreservation may vary according to species, extender composition, glycerol concentration, dose, and post-thaw evaluation time [2, 30, 31]. It is also possible that reducing mitochondrial ROS alone was insufficient to counteract the combined osmotic, membrane, acrosomal, and structural injuries induced by the freeze-thaw process. Therefore, the absence of statistically supported improvements in the MitoTEMPO groups should not be interpreted as evidence of limited biological relevance of this antioxidant, but rather as an indication that its efficacy may depend on dose, extender composition, timing of supplementation, and species-specific sperm sensitivity.

The absence of a statistically supported synergistic effect in the MitoTEMPO + PTX groups also deserves consideration. Although combining a mitochondria-targeted antioxidant with a motility-supporting agent could theoretically provide complementary protection, this was not statistically supported under the present experimental conditions. The combination groups did not provide a statistically supported additional benefit compared with trehalose or PTX at 0.5 µM alone for the main sperm quality parameters. This may reflect dose-dependent interactions between additives, changes in the balance of the extender system, or the possibility that the protective mechanisms targeted by MitoTEMPO and PTX did not act additively during cryopreservation. These findings indicate that co-supplementation should not be assumed to be beneficial without specific optimization of concentration and timing.

Several limitations of the present study should be acknowledged. First, sperm motility was evaluated by subjective microscopic assessment rather than a computer-assisted sperm analysis system. Although all motility evaluations were performed under standardized conditions by the same experienced operator who was blinded to treatment groups, CASA would provide more objective and detailed information on sperm kinematic parameters. Although motility and plasma membrane integrity are generally expected to be biologically related, they do not necessarily reflect identical aspects of sperm function. Motility may also be influenced by mitochondrial activity, ATP availability, axonemal function, oxidative stress, and post-thaw metabolic status, whereas SYBR-14/PI staining mainly reflects plasma membrane integrity and cell viability. Therefore, the lack of a closer association between these parameters should be interpreted cautiously and represents a limitation of the present study. Second, semen samples were pooled before treatment allocation. Pooling helped reduce individual ejaculate-related variation and provided sufficient semen volume for simultaneous comparison of all treatment groups under identical experimental conditions; however, it prevented the evaluation of individual ram responses and inter-animal variability. Furthermore, detailed individual breeding history and fertility records of each ram were not available, which limits the interpretation of ram-level reproductive variability. Therefore, the findings should be interpreted at the pooled-ejaculate level rather than at the individual-animal level. Finally, the present study evaluated in vitro post-thaw sperm quality and oxidative balance parameters, but did not include fertility trials. Improvements in laboratory sperm quality parameters do not necessarily translate directly into improved fertility performance. Future studies using individual ejaculates, optimized additive concentrations, CASA-based sperm motion analysis, and in vivo fertility outcomes are required before practical recommendations can be made.

## Conclusions

Trehalose and PTX at 0.5 µM significantly improved several post-thaw sperm quality traits under the conditions of this study. The results suggest that these additives differed in their statistically supported effects during cryopreservation. MitoTEMPO alone and the MitoTEMPO + PTX combinations did not provide statistically supported additional benefits for the main sperm quality parameters. Further studies including dose-response designs, objective sperm motion analysis, and fertility trials are needed to determine whether these in vitro improvements translate into reproductive benefit. 

## Data Availability

All data generated or analysed during this study are included in this published article and its tables.

## Declarations

### Prior publication

Data have not been published previously.
